# Accelerometer-derived sedentary time and physical activity and the incidence of depressive symptoms – The Maastricht Study

**DOI:** 10.1017/S0033291720004924

**Published:** 2022-10

**Authors:** Magdalena J. Konopka, Sebastian Köhler, Coen D. A. Stehouwer, Nicolaas C. Schaper, Ronald M. A. Henry, Carla J. H. van der Kallen, Hans H. C. M. Savelberg, Simone J. P. M. Eussen, Pieter C. Dagniele, Martien C. J. M. van Dongen, Miranda T. Schram, Annemarie Koster

**Affiliations:** 1 Department of Social Medicine, Maastricht University, The Netherlands; 2 School for Public Health and Primary Care (CAPHRI), Maastricht University, The Netherlands; 3 Department of Psychiatry and Neuropsychology and School for Mental Health and Neuroscience (MHeNS), Maastricht University, The Netherlands; 4 Department of Internal Medicine, Maastricht University Medical Centre, The Netherlands; 5 Cardiovascular Research Institute Maastricht (CARIM), Maastricht University, The Netherlands; 6 Heart and Vascular Centre, Maastricht University Medical Centre (MUMC+), The Netherlands; 7 Department of Human Movement Sciences, Maastricht University, The Netherlands; 8 Department of Epidemiology, Maastricht University, The Netherlands

**Keywords:** Sedentary time, Physical activity, Depressive symptoms, Accelerometer

## Abstract

**Background:**

This study examined the associations between accelerometer-derived sedentary time (ST), lower intensity physical activity (LPA), higher intensity physical activity (HPA) and the incidence of depressive symptoms over 4 years of follow-up.

**Methods:**

We included 2082 participants from The Maastricht Study (mean ± s.d. age 60.1 ± 8.0 years; 51.2% men) without depressive symptoms at baseline. ST, LPA and HPA were measured with the ActivPAL3 activity monitor. Depressive symptoms were measured annually over 4 years of follow-up with the 9-item Patient Health Questionnaire (PHQ-9). Cox regression analysis was performed to examine the associations between ST, LPA, HPA and incident depressive symptoms (PHQ-9 ⩾ 10). Analyses were adjusted for total waking time per day, age, sex, education level, type 2 diabetes mellitus, body mass index, total energy intake, smoking status and alcohol use.

**Results:**

During 7812.81 person-years of follow-up, 203 (9.8%) participants developed incident depressive symptoms. No significant associations [Hazard Ratio (95% confidence interval)] were found between sex-specific tertiles of ST (lowest *v*. highest tertile) [1.13 (0.76–1.66], or HPA (highest *v.* lowest tertile) [1.14 (0.78–1.69)] and incident depressive symptoms. LPA (highest *v.* lowest tertile) was statistically significantly associated with incident depressive symptoms in women [1.98 (1.19–3.29)], but not in men (*p*-interaction <0.01).

**Conclusions:**

We did not observe an association between ST or HPA and incident depressive symptoms. Lower levels of daily LPA were associated with an increased risk of incident depressive symptoms in women. Future research is needed to investigate accelerometer-derived measured physical activity and ST with incident depressive symptoms, preferably stratified by sex.

## Introduction

Globally, over 300 million people suffer from depression, the leading cause of disability worldwide and a major contributor to morbidity and mortality (World Health Organization, 2018). A meta-analysis of 293 studies showed that adults suffering from depression have a 1.52 times higher risk of mortality compared to non-depressed adults (Cuijpers et al., 2014). Moreover, adults with major depressive disorder have on average a 10 year lower life expectancy compared to those without depression (Walker, McGee, & Druss, 2015). Given the individual and societal burden of depression, research into determinants such as modifiable lifestyle behaviors that can prevent or lower the risk of depression are urgently needed.

It is well-established that moderate-to-vigorous physical activity (MVPA) is an important risk factor for depression (Gordon et al., 2018; Schuch et al., 2018). Consistent evidence suggests that depression rates are lowest among the most physically active fraction of the population (Teychenne, Ball, & Salmon, 2008). Furthermore, already low rates of physical activity (e.g. walking for <150 min per week) are associated with a lower incidence of depressive symptoms compared to sedentary individuals (Mammen & Faulkner, 2013). MVPA and depression are mutually related as research has shown that individuals with depression are generally less physically active than those without depression, whilst, simultaneously, lower levels of MVPA increase the risk of depression (Schuch et al., 2017). However, most evidence on the association between physical activity levels and incident depressive symptoms relies on self-reported measurements of physical activity, which are prone to bias (Schuch et al., 2018).

Next to physical activity, there is a growing interest in understanding the health effect of sedentary time (ST). Literature indicates that sitting for prolonged periods compromises overall health of adults, even in those meeting the recommendations for physical activity (Owen, Healy, Matthews, & Dunstan, 2010). ST cannot simply be defined as the absence of MVPA but should be considered as a separate domain and urges studies that evaluate its association in addition to the effects of MVPA (Dunstan, Howard, Healy, & Owen, 2012; Tremblay et al., 2017). To date, two literature reviews covering the association between ST and depressive symptoms in adults have been conducted (Teychenne, Ball, & Salmon, 2010; Zhai, Zhang, & Zhang, 2015). The studies covered by these reviews revealed inconsistent results but tentatively suggested that engaging in higher levels of daily ST is associated with a higher risk of depressive symptoms. However, the studies were mostly cross-sectional, used self-reported measurements of ST, or did not adjust for MVPA.

There is clearly a need for more research addressing existing limitations. This study captures accelerometer-derived physical activity/ST behavior and uses a longitudinal study design which minimizes the risk of reverse causation and confounding. Therefore, the aim of the current study was to examine the independent associations between accelerometer-derived ST, lower intensity physical activity (LPA), higher intensity physical activity (HPA) and the incidence of depressive symptoms during a 4-year follow-up period in a large prospective cohort study. We hypothesize that more daily ST is associated with a higher likelihood of developing depressive symptoms, independent of the amount of HPA performed. In addition, we hypothesize that less daily LPA, as well as HPA, will be associated with a higher likelihood of developing depressive symptoms.

## Materials and methods

### The Maastricht Study: population and methods

We used data from The Maastricht Study, an observational prospective population-based cohort study. The rationale and methodology have been described previously (Schram et al., 2014). In brief, the study focuses on the etiology, pathophysiology, complications and comorbidities of T2DM and is characterized by an extensive phenotyping approach. Eligible for participation were all individuals aged between 40 and 75 years living in the southern part of the Netherlands. Participants were recruited through mass media campaigns and from the municipal registries and the regional Diabetes Patient Registry via mailings. Recruitment was stratified according to known T2DM status, with an oversampling of individuals with T2DM, for reasons of efficiency. The present report includes data from the first 3451 participants, who completed the baseline survey between November 2010 and September 2013. The baseline examinations of each participant were performed within a time window of 3 months. Follow-up questionnaire data were collected annually after baseline assessments. To date, 4 years of follow-up data on depression has been available. The study has been approved by the institutional medical ethical committee (NL31329.068.10) and the Minister of Health, Welfare and Sports of the Netherlands (Permit 131088-105234-PG). All participants gave written informed consent.

For the present analyses, we excluded participants who did not receive an accelerometer due to logistics (*n* = 809). Accelerometer devices were not available during the first year of this study. Therefore, 809 random participants of the Maastricht Study had missing accelerometer data. Moreover, we excluded participants who had no baseline data of depressive symptoms (*n* = 214) and missing potential confounders (*n* = 146). In addition, we excluded participants with depressive symptoms at baseline, i.e. scoring 10 or above on the 9-item Patient Health Questionnaire (PHQ-9) (*n* = 102) and participants who had no follow-up measurements on depressive symptoms (*n* = 98), which resulted in a study population of 2082 participants. Included were participants with at least one out of four follow-up measurements.

### Accelerometer data

Daily activity levels were measured using the ActivPAL3^™^ physical activity monitor (PAL Technologies, Glasgow, UK) (Godfrey, Culhane, & Lyons, 2007). The ActivPAL3 is a small (53 × 35 × 7 mm^3^), lightweight (15g) triaxial accelerometer that records movement in the vertical, anteroposterior and mediolateral axes, and also determines posture (sitting or lying, standing and stepping) based on acceleration information. The device was attached directly to the skin on the front of the right thigh with transparent 3 M Tegaderm^™^ tape after the device had been waterproofed using a nitrile sleeve. Participants were asked to wear the accelerometer for 8 consecutive days, without removing it at any time. To avoid inaccurately identifying non-movement time, participants were asked not to replace the device once removed. Data were uploaded using the ActivPAL3 software and processed using customized software written in MATLAB R2013b (MathWorks, Natick, MA, USA). Data from the first day were excluded from the analysis because participants performed physical function tests at the research center after the device was attached. In addition, data from the final wear day providing ⩽14 wear hours of data were excluded from the analysis. Participants were included if they provided at least one valid day (⩾10 h of waking data). The total amount of ST was based on the sedentary posture (sitting or lying) and calculated as the mean time spent in a sedentary position during waking time per day. The method used to determine waking time has been described elsewhere (van der Berg et al., 2016). The total amount of stepping was based on the stepping posture and calculated as the mean time stepping during waking time per day. Stepping time (physical activity) was further classified into HPA (minutes with a step frequency >110 steps/min during waking time) and LPA (minutes with a step frequency ⩽110 steps/min during the waking time) (Tudor-Locke et al., 2011). Time spent as ST, LPA, and HPA were calculated for an average day.

### Depressive symptoms

Depressive symptoms were assessed by a validated Dutch version of the PHQ-9 (Kroenke, Spitzer, & Williams, 2001). The PHQ-9 is a self-administered questionnaire based on the Diagnostic and Statistical Manual of Mental Disorders IV criteria for a major depressive disorder (American Psychiatric Association, 2013). It comprises nine items rated on a four-point scale, ranging from 0 = ‘not at all’ to 3 = ‘nearly every day’. Both cognitive symptoms of depression, comprising thoughts about oneself and problems of the mind, as somatic symptoms of depression, comprising various bodily sensations that a depressed individual perceives as unpleasant or worrisome, are measured with the PHQ-9 (Janssen et al., 2016). Response options were used to calculate a continuous total-score ranging from 0 (no symptoms) to 27 (all symptoms present nearly every day). A pre-defined cut-off score of ⩾10 was used in the present study as a dichotomous scoring system for defining clinically relevant depressive symptoms. A PHQ-9 score ⩾10 has been associated with a sensitivity of 88% and a specificity of 88% for major depressive disorder (Janssen et al., 2016; Kroenke et al., 2001). The PHQ-9 was filled in online by the participants at baseline and annually during the follow-up time of 4 years. Lifetime depression was assessed at baseline by the Mini-International Neuropsychiatric Interview (MINI) (Sheehan et al., 1998). The MINI is a short diagnostic structured interview, used to assess the presence of minor or major depressive disorder in the past (lifetime depression) according to the DSM-IV (Diagnostic and Statistical Manual of Mental Disorders, Fourth Edition). The MINI includes questions about age at onset of first depressive episode and number of episodes.

### Covariates

The following variables were considered as potential confounders: total waking time per day, age, sex, education level, diabetes status, body mass index (BMI), total energy intake, smoking status and alcohol use. Questionnaires were used to collect information on age (in years), sex, educational level, smoking status, alcohol use and energy intake. Educational level was divided into low, middle and high. Smoking status was divided into current, former and never smokers. Alcohol consumption was divided into three categories: non-consumers, low-consumers (for women ⩽7 glasses alcohol per week; for men ⩽14 glasses alcohol per week) and high consumers (for women >7 glasses per week; for men >14 glasses alcohol per week). Energy intake was derived from a food frequency questionnaire, which was developed for The Maastricht Study and calculated as the mean energy intake per day (kcal) (van Dongen et al., 2019). To determine diabetes status, all participants (except those who use insulin or with a fasting plasma glucose >11.0 mmol/L) underwent a standardized seven-point oral glucose tolerance test after an overnight fast as described elsewhere (Schram et al., 2014). Diabetes status was defined according to the World Health Organization 2006 criteria and participants were categorized as having no diabetes, prediabetes or T2DM. Participants on diabetes medication and without type 1 diabetes were also considered as having T2DM. BMI was calculated from weight and height measured in a physical examination to the nearest of 0.5 kg or 0.1 cm.

### Statistical analysis

All analyses were performed using SPSS version 25.0 for Windows. Descriptive statistics, frequency tables and crosstabs were used to explore distribution, normality and frequency of all variables. We compared baseline characteristics of the study population between individuals with and without incident depressive symptoms. We used a *t* test for normally distributed continuous variables, a Kruskal–Wallis test for non-normally distributed continuous variables and chi-square test for categorical variables.

Cox regression analysis was used to examine the associations between ST, LPA, HPA and incident depressive symptoms, yielding hazard ratios (HRs) and 95% confidence intervals (95% CIs). Sex-specific tertiles of ST, LPA and HPA were used as independent variables since the assumption of linearity of any continuous predictor was not fulfilled. We used sex-specific tertiles in order to keep the male-female ratio in the categories equal. In survival analysis, incident depressive symptoms were treated as the failure event. Time at risk in days was calculated from study entry until study exit – the latter being date of depressive symptoms, or date of becoming lost to follow-up, or end of the study – whichever came first. Incidence density rates (IDRs) were calculated for different strata. Cox proportional hazards assumption was confirmed by testing log-time interactions and examining log-log survival plots. We considered results to be significant at *p* < 0.05 in 2-tailed tests. All analyses were adjusted for total waking time per day, sex, age, education level, diabetes status (adjusted in model 1); BMI, total energy intake, smoking status and alcohol use (additionally adjusted in model 2). Model 3 was additionally adjusted for HPA when examining SB and LPA; or for ST when examining HPA. Interaction with sex and diabetes status was tested for all models and was considered to be significant at *p* < 0.05. The models with interaction were compared to the model without interaction using likelihood ratio tests (chi-square test with 1 degree of freedom), followed by inspection of stratified effects.

Several sensitivity analyses have been performed. In sensitivity analysis 1, participants with complete follow-up data of depressive symptoms were analyzed (*n* = 1397). In sensitivity analysis 2, participants with at least three follow-up measurements were included in the analysis (*n* = 1814); and in sensitivity analysis 3, participants with at least 4 days of valid ActivPAL3 data were selected (*n* = 1996). In sensitivity analysis 4, we additionally adjusted for the use of antidepressants in Model 2. In sensitivity analysis 5, we excluded participants with lifetime depressive episodes based on the MINI (*n* = 1403). In sensitivity analysis 6, we additionally adjusted for lifetime depression in Model 2. In sensitivity analysis 7 and 8, level of education was replaced by income (low, middle, high) and occupation status (employed, unemployed), respectively. In sensitivity analysis 9, BMI was replaced by waist circumference. Finally, we also analyzed the cross-sectional association between ST, LPA, HPA and depressive symptoms at baseline (PHQ-9) using logistic regression analysis yielding odds ratios (ORs).

## Results

The original sample consisted of 3451 participants, of whom 975 (28.3%) had T2DM. The current study population consisted of 2082 participants, of whom 528 (25.4%) had T2DM. Table 1 presents the baseline characteristics of the study population stratified by incident depressive symptoms. The participants, of whom 51.2% were men, had a mean ± s.d. age of 60.1 ± 8.0 years. The median follow-up time was 4.1 ± 0.9 years. Of the 2082 participants, 203 (9.6%) developed depressive symptoms (PHQ-9 ⩾ 10) during the 7812.81 person-years of follow-up. The estimated IDR was 25.98 cases per 1000 person-years. Included participants provided on average 4.8 ± 2.8 valid days of accelerometry data, although most participants (56.4%) provided 7 valid days. Mean time spending awake during the day was 943.9 ± 54.3 min, of which 561.2 ± 98.1 min were spent sedentary. At baseline, participants who developed incident depressive symptoms spent per day, on average, 18.1 min more sedentary, 8.1 min performing less LPA and 5.2 min performing less HPA, compared to those who did not develop depressive symptoms. In comparison to the included study population, those who were excluded (*n* = 1369) were on average older, had a lower level of education, a higher BMI, were more often smokers and had a higher level of ST, lower level of LPA and HPA, respectively (Table 1, appendix). We also compared the baseline characteristics of those with complete follow-up data to those with one or more missing follow-up measurements (Table 2, appendix). No differences in ST and incident depressive symptoms were observed. Participants with complete follow-up data had only slightly higher levels of LPA and HPA compared to those with missing follow-up data.

**Table 1.** Baseline characteristics of the study population stratified by incident depressive symptoms measured with the 9-item Patient Health Questionnaire (PHQ-9 ⩾ 10)

	Total	No depressive symptoms	Depressive symptoms	*P* value
Total population, *n* (%)	2082 (100.0)	1879 (90.2)	203 (9.8)	
Age (years), mean (s.d.)	60.1 (8.0)	60.1 (8.0)	59.7 (8.0)	0.46
Sex, n (%)				0.99
Men	1067 (51.2)	963 (51.3)	104 (51.2)	
Women	1015 (48.8)	916 (48.7)	99 (48.8)	
Education, n (%)				<0.01
Low	648 (31.1)	562 (29.9)	86 (42.4)	
Medium	594 (28.5)	531 (28.3)	63 (31.0)	
High	840 (40.3)	786 (41.8)	54 (26.6)	
BMI (kg/m^2^), mean (s.d.)	26.8 (4.3)	26.6 (4.2)	28.4 (5.3)	<0.01
Energy intake, (kcal/day), mean (s.d.)	2161.2 (586.2)	2155.5 (583.6)	2213.7 (609.2)	0.18
Smoking, n (%)				<0.01
Never	758 (36.4)	701 (37.3)	57 (28.1)	
Former	1088 (52.3)	983 (52.3)	105 (51.7)	
Current	236 (11.3)	195 (10.4)	41 (20.2)	
Alcohol (drinks/week), *n* (%)				0.01
None	325 (15.6)	280 (14.9)	45 (22.2)	
Moderate	1201 (57.7)	1079 (57.4)	122 (60.1)	
High	556 (26.7)	520 (27.7)	36 (17.7)	
Diabetes status, n (%)				<0.01
No diabetes	1212 (58.2)	1123 (59.8)	89 (43.8)	
Prediabetes	319 (15.3)	293 (15.6)	26 (12.8)	
Type 2 diabetes	528 (25.4)	446 (23.7)	82 (40.4)	
Other type	23 (1.1)	17 (0.9)	6 (3.0)	
Antidepressants, n (%)				<0.01
No	1966 (94.4)	1793 (95.4)	173 (85.2)	
Yes	116 (5.6)	86 (4.6)	30 (14.8)	
Waking time (min/day), mean (s.d.)	943.9 (54.3)	944.9 (54.1)	934.1 (55.4)	0.01
ST (min/day), mean (s.d.)	561.2 (98.1)	559.5 (96.6)	577.6 (109.4)	0.01
LPA (min/day), mean (s.d.)	98.1 (32.6)	98.9 (32.1)	90.8 (36.5)	<0.01
HPA (min/day), median (IQR)	19.2 (9.6–32.2)	19.6 (9.9–32.5)	14.4 (5.7–28.3)	<0.01

BMI = body mass index, ST = sedentary time, LPA = lower intensity physical activity, HPA = higher intensity physical activity.

s.d. = standard deviation, IQR = interquartile range.

**Table 2.** Associations between sedentary time (ST), lower intensity physical activity (LPA), higher intensity physical activity (HPA) and incident depressive symptoms measured with the 9-item Patient Health Questionnaire (PHQ-9 ⩾ 10)

	Events, n(%)	Person-years	Incidence rate (per 1000 py)	Model 1		Model 2		Model 3	
				HR	95% CI	HR	95% CI	HR	95% CI
ST*									
1 (lowest)	57 (8.2)	2594	21.97	REF		REF		REF	
2 (middle)	72 (10.4)	2618	27.50	1.32	0.93–1.87	1.28	0.90–1.81	1.25	0.87–1.78
3 (highest)	74 (10.7)	2600	28.46	1.34	0.93–1.93	1.18	0.81–1.71	1.13	0.76–1.66
LPA**									
1 (highest)	54 (7.8)	2642	20.44	REF		REF		REF	
2 (middle)	58 (8.3)	2612	22.20	1.06	0.73–1.54	1.05	0.72–1.54	1.04	0.72–1.52
3 (lowest)	91 (13.1)	2558	35.67	1.41	1.00–2.00	1.26	0.88–1.80	1.23	0.85–1.77
HPA***									
1 (highest)	53 (7.6)	2667	19.87	REF		REF		REF	
2 (middle)	57 (8.2)	2622	21.74	1.02	0.70–1.48	0.94	0.65–1.38	0.91	0.62–1.33
3 (lowest)	93 (13.4)	2524	36.84	1.49	1.04–2.13	1.25	0.86–1.81	1.14	0.78–1.69

HR = hazard ratio, 95% CI = 95% confidence interval, py = person-years.

Model 1 is adjusted for sex, age, level of education, diabetes status and total waking time (h).

Model 2 is additionally adjusted for body mass index, smoking, alcohol and total energy intake.

Model 3 describing ST and LPA is additionally adjusted for HPA (h) and model 3 describing HPA is additionally adjusted for ST (h).

*Mean (s.d.) ST men: lowest 487.8 (51.7) min/d, middle 539.5 (23.5) min/d, highest 693.1 (49.6) min/d.

*cut off value ST men: lowest-middle tertile 553.5 min/d, middle-highest tertile 634.9 min/d.

*Mean (s.d.) ST women: lowest 430.7 (50.8) min/d, middle 528.2 (23.2) min/d, highest 629.1 (48.3) min/d.

*cut off value ST women: lowest-middle tertile 487.7 min/d, middle-highest tertile 567.5 min/d.

**Mean (s.d.) LPA men: lowest 61.5 (12.9) min/d, middle 95.2 (9.7) min/d, highest *138.6* (23.9) min/d.

**Cut off value LPA men: lowest-middle tertile 78.5 min/d, middle-highest tertile 111.7 min/d.

**Mean (s.d.) LPA women: lowest 68.1 (11.3) min/d, middle 95.3 (6.5) min/d, highest *130.0* (20.9) min/d.

**Cut off value LPA women: lowest-middle tertile 84.1 min/d, middle-highest tertile 106.8 min/d.

***Mean (s.d.) HPA men: lowest 5.0 (2.4) min/d, middle 14.8 (3.8) min/d, highest *39.5* (16.8) min/d.

***Cut off value HPA men: lowest-middle tertile 9.1 min/d, middle-highest tertile 22.4 min/d.

***Mean (s.d.) HPA women: lowest 10.3 (4.3) min/d, middle 23.6 (4.1) min/d, highest *47.4* (17.1) min/d.

***Cut off value HPA women: lowest-middle tertile 17.2 min/d, middle-highest tertile 31.2 min/d.

Table 2 presents the results of the Cox regression analysis with ST, LPA and HPA as exposures and incident depressive symptoms as an outcome. No significant associations were found between sex-specific tertiles of ST and the incidence of depressive symptoms (Model 3, highest *v.* lowest sex-specific tertile; HR 1.13; 95% CI 0.76–1.66). LPA was statistically significantly associated with an increased risk of developing depressive symptoms in Model 1 (lowest *v.* highest sex-specific tertiles; HR 1.41; 95% CI 1.00–2.00). However, when additionally adjusted for covariates in Model 2 and 3 the associations were no longer statistically significant (Model 3, HR 1.23; 95% CI 0.85–1.77). In Model 1, compared to those in the highest sex-specific tertile of HPA, those in the lowest sex-specific tertile had a statistically significantly increased risk of developing depressive symptoms (HR 1.49; 95% CI 1.04–2.13). After adjustment for additional covariates in Model 2 and 3 the associations between HPA and the incidence of depressive symptoms were no longer statistically significant (Model 3, HR 1.14, 95% CI 0.78–1.69).

Interaction between ST, LPA, HPA and sex and diabetes status was tested for all models. The interaction with diabetes status was not statistically significant for ST (*p* = 0.80), LPA (*p* = 0.41), HPA (*p* = 0.51) and incident depressive symptoms. Sex was a significant effect modifier for LPA (Model 3, Wald: 10.16; df: 2; *p* value: 0.006); the stratified results are shown in Table 3. Compared to women in the highest tertile of LPA, those in the lowest tertile had a statistically significantly increased risk of developing depressive symptoms (Model 3, HR 1.98; 95% CI 1.19–3.29). In contrast, we did not find a significant association between LPA and the incidence of depressive symptoms in men.

**Table 3.** Associations between lower intensity physical activity (LPA) and incident depressive symptoms measured with the 9-item Patient Health Questionnaire (PHQ-9 ⩾ 10) stratified by sex

	Events, n (%)	Person-years	Incidence rate (per 1000 py)	Model 1		Model 2		Model 3	
				HR	95% CI	HR	95% CI	HR	95% CI
LPA Men									
1 (lowest)	30 (8.5)	1363	22.01	REF		REF		REF	
2 (middle)	35 (9.8)	1312	26.68	1.08	0.66–1.78	1.02	0.62–1.69	1.01	0.61–1.68
3 (highest)	39 (11.0)	1336	29.19	0.91	0.55–1.50	0.74	0.44–1.25	0.73	0.43–1.25
LPA Women									
1 (highest)	24 (7.1)	1280	18.75	REF		REF		REF	
2 (middle)	23 (6.8)	1301	17.68	0.95	0.53–1.70	0.98	0.55–1.76	0.97	0.54–1.73
3 (lowest)	52 (15.4)	1222	42.55	2.16	1.32–3.56	2.05	1.24–3.39	1.98	1.19–3.29

HR = hazard ratio, 95% CI = 95% confidence interval, py = person-years.

Model 1 is adjusted for age, level of education, diabetes status and total waking time (h).

Model 2 is additionally adjusted for body mass index, smoking, alcohol and total energy intake.

Model 3 is additionally adjusted for HPA.

Several sensitivity analyses were performed. In sensitivity analysis 1, we only included participants with complete follow-up data (*n* = 1397). During the 5706.88 person-years, 126 (9.0%) participants developed incident depressive symptoms. Similar results were found as in the main analysis. No significant associations between sex-specific tertiles of ST, LPA or HPA and the incidence of depressive symptoms were observed (results not tabulated). Next, in sensitivity analysis 2, participants with at least three follow-up measurements were included in the analysis (*n* = 1814). During the 7138.42 person-years, 203 (11.2%) participants developed incident depressive symptoms. Again, no significant associations were found between sex-specific tertiles of ST or HPA and incident depressive symptoms (results not tabulated). In sensitivity analysis 3, we analyzed participants with at least 4 days of valid ActivPAL3 data (*n* = 1996), which also yielded similar results (results not tabulated). In sensitivity analysis 4, we additionally adjusted for antidepressants in Model 2 and in sensitivity analysis 5, we excluded participants whoever had depressive episodes (*n* = 1403). In sensitivity analysis 6, we additionally adjusted for lifetime depression in Model 2. Again, in sensitivity analysis 4, 5 and 6, no significant associations were found between sex-specific tertiles of ST, LPA or HPA and incident depressive symptoms (results not tabulated). Replacing level of education with income or employment status, respectively (sensitivity analysis 7 and 8), did not change the main findings (results not tabulated). Lastly, replacing BMI by waist circumference (sensitivity analysis 9) did also not change the main findings (results not tabulated). In all sensitivity analysis, except in sensitivity analysis 5, women in the lowest tertile of LPA had a statistically significant increased risk of developing depressive symptoms compared to women in the highest tertile. Finally, the cross-sectional analysis (*n* = 2241) resulted in statistically significant associations for HPA and baseline depressive symptoms (Model 3, lowest *v.* highest sex-specific tertile; OR 2.17; 95% CI 1.04–4.56) (Appendix, Table 3).

## Discussion

In this study, we examined the independent associations between accelerometer-derived ST, LPA, HPA and the incidence of depressive symptoms during a follow-up time of 4 years. After adjustment for covariates, the results showed no significant associations between ST or HPA and incident depressive symptoms in the total sample. However, low LPA was associated with an increased risk of developing depressive symptoms compared to high LPA in women, but not in men.

In contrast to our findings, most previous studies showed that lower levels of ST, higher levels of LPA and HPA, were significantly associated with an increased risk of incident depressive symptoms (Mammen & Faulkner, 2013; Schuch et al., 2018; Teychenne et al., 2008; Teychenne et al., 2010; Zhai et al., 2015). The studies often differ in study designs, follow-up lengths, study populations and sample sizes, which makes it difficult to compare their results. For example, a recent meta-analysis of 49 prospective cohort studies showed that, compared to adults engaging in lower levels of physical activity, those who engage in higher levels of physical activity had lower odds for developing depressive symptoms (pooled OR 0.83; 95% CI 0.79–0.88) (Schuch et al., 2018). Although this indicates an inverse association, most studies assessed physical activity by using self-reported questionnaires, and merely one of the studies in this meta-analysis used accelerometer-derived physical activity data (Hiles et al., 2015). That study among 1410 adults aged 55–85 years did not observe a significant association between physical activity and incident depressive symptoms (OR women 1.03; 95% CI 0.92–1.15; OR men 1.04; 95% CI 0.93–1.17). In addition, another review of prospective studies from 2013 with different in – and exclusion criteria than the more recent meta-analysis by Schuch et al. (2018) showed that four out of 30 articles did find an association between physical activity and depressive symptoms specific for women – which is in line with our results for LPA (Mammen & Faulkner, 2013). However, again, only one study used accelerometer-derived physical activity data (Åberg et al., 2012), which underlines the need for future studies with accelerometer-derived physical activity data.

Our study did not find an overall protective effect of physical activity on the onset of depressive symptoms; however, we did observe a protective effect for LPA in women. The discrepant results between our study and others might be explained by differences in the collection of physical activity data, as well as in the assessment of depressive symptoms. Studies using self-reported physical activity data may be subject to recall bias and consequently may contain information bias, which would most likely result in differential misclassification of physical activity data (Althubaiti, 2016). Adults suffering from depressive symptoms might have a more negative self-reflection compared to adults who do not suffer from depressive symptoms (Scott & O'Hara, 1993). Those adults might estimate their daily physical activity level as less active compared to those without depressive symptoms. As a consequence, this could have led to an overestimation of the associations found between self-reported levels of physical activity and incident depressive symptoms in previous studies.

It seems that the effect of physical activity levels and incident depressive symptoms differ between men and women. It has been suggested that women might benefit from the cognitive-psychological aspects of physical activity more than men, for example acquiring more self-esteem (Mikkelsen et al., 2010). In comparison, men might be more active during work-time, which has been associated with an increased risk of incident depressive symptoms (Baumeister et al., 2017; McKercher et al., 2009). In the current study, we were not able to differentiate between occupational and leisure-time physical activity levels.

Two literature reviews that have been conducted on the association between ST and depression revealed inconsistent results, but tentatively suggested that engaging in higher levels of ST is associated with an increased risk for depression (Teychenne et al., 2010; Zhai et al., 2015). However, of the two reviews, only three studies had accelerometer-derived ST and only one of those had a longitudinal study design (Sanchez-Villegas et al., 2008). In line with our results, this prospective study did also not find any association between accelerometer-derived ST, or any association between physical activity and depression. Moreover, a recently performed longitudinal study in older adults (age > 65 years) with a follow-up time of 2 years did report an association between accelerometer-derived LPA, independent of the amount MVPA and the incidence of depressive symptoms. However, ST and MVPA were not independently associated with incident depressive symptoms in this study (Ku, Steptoe, Liao, Sun, & Chen, 2018). The authors of this study explained the discrepancy of their findings between LPA and MVPA as possibly indicating that engaging in physical activity with moderate-to-vigorous intensity might produce discomfort and shortness of breath, especially in older adults and thus does not help to prevent developing depressive symptoms, in contrast to LPA. To our knowledge, only two longitudinal studies with accelerometer-derived ST and the risk of depression have been conducted so far, both with statistically insignificant results (Ku et al., 2018; Sanchez-Villegas et al., 2008). Just one of the two studies did statistically adjust for MVPA as a covariate (Ku et al., 2018).

We would like to emphasize several strengths of our analyses. First, the use of tri-axial accelerometers in a large sample size allows the objective measurement of ST and physical activity data, while most other studies used self-reported questionnaires prone to information bias. The major advantage of the ActivPAL3 is that it distinguishes changes in posture based on precise acceleration information so that ST and physical activity can be measured more accurately than other monitors. Second, the longitudinal study design takes temporality into account, one of the requirements for causality (Rothmann & Greenland, 2005).

Nonetheless, this study is not without limitations. First, we used a pre-defined cut-off score of PHQ9⩾10 for defining clinically relevant depressive symptoms and did not use clinically diagnosed major depressive disorder. However, the PHQ-9 was found to have acceptable diagnostic properties for detecting major depressive disorder for cut-off scores between eight and 11, and it is one of the most used questionnaires in epidemiological studies to determine clinically relevant depressive symptoms (Janssen et al., 2016; Manea, Gilbody, & McMillan, 2012). Furthermore, we excluded participants scoring 10 or higher on the PHQ-9 at baseline (*n* = 102). Including participants with sub-threshold depressive symptoms leaves the door open for reverse causation. For instance, participants who scored a nine on the PHQ-9 at baseline are included in our analysis. Those participants could have changed their physical activity behavior due to the presence of sub-threshold depressive symptoms. In additional analyses, we adjusted for lifetime depression and excluded participants with lifetime depression which did not change our results. In the analyses in which participants with lifetime depression were excluded (*n* = 625), the interaction between LPA and sex was no longer significant which might be due to lack of power as the number of participants with incident depressive symptoms was considerably lower. Second, it might be possible that the follow-up questionnaires to determine depressive symptoms were filled in more often by non-depressed individuals and less often by participants having severe depressive symptoms, due to lack of motivation by depressed individuals, leading to loss of power (fewer incident cases). For this reason, we conducted several sensitivity analyses, one with complete follow-up data, which did not influence our main findings indicating the robustness of our findings. Third, 1369 participants were excluded from the main analysis due to missing data. The included study population was a healthier selection compared to the excluded one (Table 1, appendix). Furthermore, the incidence of depressive symptoms did not differ in those with one or more missing follow-up measure compared to participant with complete follow-up data (Table 2, appendix). Fourth, our study population consisted of a highly functioning population aged 40–75 years of predominantly Caucasians from European descent which should be considered when generalizing our results to other populations. Fifth, the Maastricht Study was for reasons of efficiency enriched with participants with T2DM. We therefore adjusted all analyses for T2DM. Moreover, we tested interaction with T2DM which was not statistically significant. Finally, we analyzed associations with HPA based on step-frequency which may be less precise than using acceleration data to determine intensity levels.

The results of this project contribute to the understanding of the role between ST and physical activity and the risk of depressive symptoms. In conclusion, our results indicate, that lower levels of daily LPA are associated with an increased risk of developing depressive symptoms in women. Further research with a longitudinal design, accelerometer-derived ST and physical activity data, stratified by sex, is necessary to extend existent evidence for the prevention of depressive symptoms by reducing ST or increasing physical activity for those at high risk. Moreover, an interesting topic for future research would be to differentiate between accelerometer-derived leisure-time and occupational physical activity and ST, and their association with incident depressive symptoms.
